# Cerebrospinal fluid metagenomic next-generation sequencing for culture-negative anaerobic central nervous system infection involving *Prevotella oris*: attribution challenges in low-biomass specimens

**DOI:** 10.3389/fimmu.2026.1896093

**Published:** 2026-10-12

**Authors:** Yan Li, Minghua Fan, Yuyuan Hu, Yuhang Yang, Wenyuan Li, Wei Zhang, Yanhong Wang

**Affiliations:** 1 Neurology, Third Hospital of Shanxi Medical University, Shanxi Bethune Hospital, Shanxi Academy of Medical Sciences Tongji Shanxi Hospital, Taiyuan, China; 2 Division of Colorectal Surgery, Third Hospital of Shanxi Medical University, Shanxi Bethune Hospital, Shanxi Academy of Medical Sciences Tongji Shanxi Hospital, Taiyuan, China; 3 Yuncheng Central Hospital affiliated to Shanxi Medical University, Yuncheng, China

**Keywords:** anaerobic meningitis, cerebrospinal fluid, clinical attribution, host response, low-biomass specimens, metagenomic next-generation sequencing, *Prevotella oris*

## Abstract

Anaerobic central nervous system (CNS) infections are uncommon but diagnostically difficult because fastidious organisms may be suppressed by empirical antimicrobial therapy and conventional cultures may remain negative. Cerebrospinal fluid (CSF) metagenomic next-generation sequencing (mNGS) can recover microbial nucleic acids in this setting, but low microbial biomass creates a central attribution problem: a detected organism may represent active infection, introduced commensal DNA, laboratory background, or bioinformatic misclassification. This narrative Mini Review uses *Prevotella oris* as a representative oral anaerobe to examine that problem. We synthesize evidence on low-biomass CSF specimen processing and sequencing strategies, including nanopore sequencing, whole-genome amplification, and targeted approaches, alongside contamination control, sequence-level and taxonomic credibility, host-response immunology, neuroimaging and source concordance, antimicrobial timing, and discordant clinical scenarios. We then organize these established principles into an expert-informed two-step pathway that separates technical signal credibility from clinical attribution and explicitly preserves an indeterminate state when evidence conflicts. *P. oris* is positioned as a useful model of the plausible contaminant paradox rather than as a pathogen with a unique CNS phenotype. We also outline syndrome-based management, source control, and treatment-duration principles. The framework is conceptual and non-validated; its purpose is transparent reasoning and reporting, not diagnostic scoring or probability estimation.

## 1 Introduction

Anaerobic central nervous system (CNS) infections are uncommon but clinically important because microbiological confirmation is often difficult after empirical antimicrobial exposure and because oral anaerobes may reach the CNS by dental, sinus, otogenic, or other contiguous head-and-neck routes (1–4). Cerebrospinal fluid (CSF) metagenomic next-generation sequencing (mNGS) can recover pathogen nucleic acids when routine testing is unrevealing, but the interpretative problem is particularly acute in CSF because microbial biomass is usually low and background DNA can become proportionally prominent (1, 2, 5–8). Thus, detection and attribution are not equivalent: a microbial readout may be technically real yet clinically irrelevant, or clinically important despite a weak signal after pretreatment.

Oral anaerobes illustrate this tension especially well. *Prevotella*, *Fusobacterium*, *Porphyromonas*, *Parvimonas*, and related taxa are repeatedly identified in intracranial suppurative infection, often in polymicrobial communities (3, 4, 9, 10). At the same time, oral-associated DNA detected at low abundance in a contamination-sensitive CSF specimen can be difficult to distinguish from introduced or background signal. We use the term “plausible contaminant paradox” for this circumstance: the same organism is biologically plausible as a CNS pathogen and analytically plausible as background.

This narrative Mini Review uses *Prevotella oris* as a representative model to examine that attribution problem. We first consider how specimen processing and sequencing strategy shape the signals available for interpretation, and then apply the two-step framework to technical credibility and clinical attribution. Rather than proposing a new diagnostic algorithm, we synthesize established laboratory and clinical principles into an expert-informed, non-validated pathway that first asks whether the microbial signal is technically credible and then asks how strongly it can be attributed to active CNS infection. The intended contribution is organizational: to make uncertainty explicit, preserve an indeterminate state when evidence is discordant, and link interpretation to transparent reporting rather than fixed numerical thresholds or implied infection probabilities.

## 2 Literature search and review approach

This is a narrative Mini Review rather than a systematic review. PubMed/MEDLINE was searched from database inception through 19 August 2026 using combinations of terms spanning: (i) CSF mNGS and low-biomass interpretation (“metagenomic next-generation sequencing”, “cerebrospinal fluid”, “low biomass”, “contamination”, “negative controls”, “genome coverage”, and “taxonomic classification”); (ii) anaerobic CNS syndromes (“central nervous system infection”, “meningitis”, “encephalitis”, “brain abscess”, and “subdural empyema”); (iii) *P. oris* and other oral anaerobes (“*Prevotella oris*”, “*Prevotella*”, “*Fusobacterium*”, “*Porphyromonas*”, and “oral anaerobes”); (iv) host-response immunology (“host response”, “transcriptomics”, “proteomics”, “cytokines”, and “chemokines”); and (v) clinical management (“antimicrobial therapy”, “anaerobic coverage”, “source control”, “treatment duration”, and “guideline”). No formal language filter was applied at the database-search stage. We prioritized peer-reviewed clinical studies, analytical or validation studies, current guidance documents, and informative case reports or case series that directly addressed CSF mNGS, low-biomass signal interpretation, anaerobic CNS infection and oral anaerobes, host-response evidence, or syndrome-specific antimicrobial and source-control management. The search was intended to support a focused narrative synthesis; no PRISMA-based selection process, formal risk-of-bias assessment, or meta-analysis was undertaken. For this revision, a focused supplementary PubMed/MEDLINE search was performed on 28 September 2026 using “cerebrospinal fluid” or “CSF” in combination with terms for nanopore or MinION sequencing, whole-genome or multiple-displacement amplification, and targeted metagenomic sequencing, 16S amplicons, or target capture. Relevant publications appearing after the original 19 August 2026 search date were considered.

## 3 From detection to attribution in low-biomass CSF

Clinical mNGS studies show both the value and the limitations of agnostic sequencing. In a multicenter prospective study of 204 patients with meningitis or encephalitis, CSF mNGS identified 13 of 58 diagnosed CNS infections that had not been detected by testing at the referring hospitals, but 26 clinically diagnosed infections were missed because diagnosis depended on serology, non-CSF tissue, or pathogen titers below the assay detection range (2). Brain-abscess cohorts demonstrate a more pronounced advantage for sequencing in anaerobic and polymicrobial disease. In a 10-year retrospective cohort, mNGS positivity exceeded culture in both pus (85.0% vs. 50.0%) and CSF (84.2% vs. 7.9%), and anaerobes were detected in 18.4% of CSF samples by mNGS compared with none by culture (3). A separate 2026 cohort likewise reported mixed infections in 53.2% of mNGS-tested cases and higher anaerobe detection with mNGS than culture (50.0% vs. 16.7%) (4). These studies consistently demonstrate the added microbiological yield of mNGS in brain abscess, but their predominantly suppurative-disease populations should not be used to derive attribution thresholds for low-biomass CSF in isolated meningitis.

### 3.1 Pre-analytical and sequencing strategies for low-biomass CSF

In low-biomass CSF, the signal available for later interpretation depends on specimen processing, nucleic-acid recovery, microbial lysis, host background, and the controls carried through extraction and library preparation (8, 11). These upstream choices should therefore be documented before read abundance, taxonomic assignment, or sequence distribution is interpreted.

Nanopore sequencing can provide long reads and streaming analysis, but its clinical performance depends on the library strategy and patient population (12, 13). Several CSF studies amplified bacterial 16S rRNA sequences before nanopore sequencing; their findings inform broad-range bacterial amplicon testing and should not be presented as the performance of untargeted shotgun mNGS (12, 14, 15). In a small pilot study, additional detections occurred alongside discordant results and background-noise concerns, while its short analysis interval did not represent the entire specimen-to-report workflow (12).

Whole-genome amplification can make scarce extracted DNA available for sequencing and has provided additional genomic information in a proof-of-concept study of CSF shunt infection (16). It is not equivalent to selective pathogen enrichment: a CSF workflow comparison found little relative enrichment of pathogen reads with WGA alone in the absence of host depletion (11). Amplified read abundance and sequence distribution consequently require interpretation against process-matched controls and validation of the particular workflow, rather than being treated as direct measures of the starting microbial burden.

Targeted approaches include bacterial 16S amplicon sequencing and probe-based capture of predefined sequences (15, 17). In a postoperative CSF cohort, 16S nanopore sequencing identified bacterial signals in some culture-negative samples (15); a target-capture study found potential pathogens in 8 of 18 conventionally negative suspected infections, 7 of which were confirmed by qPCR (17). These findings suggest utility in selected settings, but primer and probe design restrict the detectable spectrum, and negative results cannot exclude off-target organisms (15, 17). The capture study also reported 59% positive agreement with conventional diagnostics in confirmed infections and many unexpected detections, so additional signals require control-aware clinical assessment (17). Read abundance and sequence distribution should be interpreted with assay-specific evidence rather than compared directly with untargeted shotgun data (15, 17). Thus, the framework below evaluates technical credibility using evidence appropriate to the assay before considering independent host-response and anatomic-clinical evidence.

### 3.2 Laboratory signal credibility

Low biomass amplifies the relative impact of exogenous DNA. Salter et al. demonstrated that reagent and laboratory contaminants can dominate sequence profiles as starting microbial biomass falls (5), while statistical approaches such as decontam use DNA concentration and negative-control prevalence to identify likely contaminants (6). In low-biomass 16S rRNA sequencing experiments, blanket removal of taxa detected in negative controls also removed expected community members; this finding cautions against treating control overlap as an automatic veto, but it does not establish a cutoff for clinical CSF mNGS (7). CSF mNGS studies instead support examining the patient signal alongside concurrently processed controls, background patterns, and the specificity and distribution of mapped reads, using criteria validated for the laboratory workflow (8, 18). CSF-specific studies therefore favor composite assessment of control profiles, mapping characteristics, sequence diversity, and background rather than binary control subtraction (8, 18, 19).

For practical interpretation, “dominance” is best treated as relative prominence among non-host microbial reads within the same specimen, ideally using a locally validated normalized metric rather than a universal percentage. Enrichment should likewise be judged against concurrently processed controls and batch-specific background; a laboratory may use validated RPM/RPTM, fold-enrichment, z-score, or background-percentile approaches, but one cutoff should not be transferred across platforms (8, 18, 19). For genome-wide shotgun data with sufficient informative reads, coherent sequence evidence includes mapping across multiple distinct, nonrepetitive genomic regions rather than clustering in low-complexity or nonspecific loci (8, 18). Whole-genome distribution cannot be assessed from a restricted 16S amplicon; such results require locus-appropriate checks of sequence specificity and control background (12, 15). Low-level detection of the same taxon in a negative control lowers confidence but does not automatically invalidate a substantially enriched patient signal (7). Differences in sequencing depth favor normalized rather than raw-read comparisons; recurrent laboratory taxa should be interpreted against a maintained batch/background profile; and species-level calls should be downgraded when unique or diagnostic alignments are insufficient to exclude near-neighbor misclassification (8, 18–20). **Supplementary Table 2** summarizes these principles together with assay and library design. Laboratories should use workflow-validated normalized metrics, process-matched controls, and sequence-level evidence available from the assay; genome-wide distribution criteria apply only when the workflow generates genome-wide data, and no universal threshold should be transferred across platforms or enrichment strategies (8, 11, 17).

### 3.3 Host-response immunology

Host-response evidence addresses a different question from read-level quality: whether the patient has a biologically compatible CNS inflammatory process. In studied bacterial meningitis populations, paired CSF and serum measurements have shown intrathecal enrichment of TNF and IL-6, and CSF inflammatory cells have shown NF-κB activation (21, 22). Elevated CSF chemokines and antibody-neutralization experiments support a contribution of IL-8 and GRO-α to *in vitro* CSF-mediated neutrophil chemotaxis, whereas complement responses differ between pneumococcal and meningococcal meningitis (23, 24). Intrathecal TNF has also been associated with the CSF-to-serum albumin quotient, a measure of barrier dysfunction (25). These observations make an inflammatory CSF profile biologically informative, but they do not identify the organism responsible or establish a *P. oris*-specific mechanism.

Routine leukocyte count and differential, protein, glucose, and CSF-to-serum glucose ratio remain rapid but nonspecific markers. Research assays provide a more detailed view of the host response, but their demonstrated classification tasks must be specified. CSF IL-6 and IL-10 were evaluated for distinguishing pediatric bacterial meningitis from selected comparators, while proteomic profiles separated broader meningitis etiologies rather than individual oral-anaerobe species (26, 27). Combined microbial sequencing and host-transcriptomic approaches have been studied in defined tuberculosis-meningitis and pediatric meningitis/encephalitis cohorts (28, 29). For the present attribution problem, such findings can support assessment of inflammation or a broad infection class; they have not validated identification of *P. oris* as the cause of an individual CSF signal.

### 3.4 Clinical concordance, discordance, and source plausibility

Clinical attribution should integrate the technically interpretable microbial signal with syndrome, anatomy, host response, source plausibility, antimicrobial timing, and longitudinal course (20, 30). Discordance in one domain should trigger reassessment rather than automatic exclusion. In a retrospective bacterial-meningitis series, meningitis-associated MRI abnormalities were identified in 62 of 75 imaged patients; the absence of such findings in the remaining patients shows that a non-diagnostic MRI cannot, by itself, exclude meningitis (31). This observation does not establish the sensitivity of MRI for early disease, focal anaerobic infection, or *P. oris* specifically. The reverse discordance also occurs: 39 of 2,357 episodes in a nationwide cohort of bacterial meningitis presented with a normal CSF leukocyte count, particularly among immunocompromised patients (32). Antibiotic pretreatment further complicates interpretation; CSF mNGS remained more sensitive than culture in purulent meningitis after antibiotics, but detection declined with longer treatment exposure (33).

When CSF is inflammatory and an oral-anaerobe signal is technically credible, but the initial MRI shows no meningitis-associated or focal finding, the imaging result should not end the infection assessment. Reassess sampling and antimicrobial timing, the organism-specific mapping and controls, and evidence of a dental, sinus, otogenic, or other source; obtain follow-up imaging when the clinical course continues to suggest focal infection (18, 31, 33). Conversely, convincing abscess or empyema imaging with little CSF pleocytosis warrants review of immune status and treatment timing and, when clinically feasible, microbiological assessment of the lesion rather than automatic exclusion based on lumbar CSF findings (32, 34). If the same taxon occurs in patient and control libraries, compare normalized enrichment, assay-appropriate sequence specificity or genome distribution, and recurrence across the batch before assigning descriptive attribution; unresolved conflict should remain indeterminate (7, 18). These are targeted reassessment steps, not validated decision rules.

## 4 *P. oris* as a model and the proposed attribution framework

*P. oris* is selected as an illustrative organism because oral-site detection and rare CNS case reports make infection biologically plausible, while neither its presence in CSF nor its species label resolves the low-biomass attribution problem. A species-specific PCR study detected P. oris in 17 of 47 dentoalveolar abscess specimens and in three specimens from spreading odontogenic infections (35). Published CNS reports include culture-negative meningitis with spinal canal infection and CSF mNGS detection of *P. oris*, and a sinus-associated intracranial infection with P. oris and another oral-anaerobe signal in CSF (36, 37). These case-level observations support clinical plausibility but do not establish incidence, a distinctive CNS phenotype, or monomicrobial causality. Brain-abscess sequencing studies identify polymicrobial infections involving oral-associated anaerobes, including *Prevotella*, Fusobacterium, and Porphyromonas, whereas directly informative *P. oris* CNS evidence remains case based (3, 9, 36, 37). No validated combination of symptoms, routine CSF findings, imaging, presumed source, or response to broad antimicrobial therapy distinguishes *P. oris* from another oral anaerobe. Source and anatomy inform whether an oral-anaerobe route is plausible; species identity requires adequate taxonomic evidence or orthogonal confirmation where available, and species-level causality remains a separate clinical judgment (2).

### 4.1 What the framework adds

Several components of interpretation are already established. Wilson et al. used control-derived weighted z scores to prioritize mNGS taxa (19); Wang et al. combined clinical, conventional microbiological, and CSF inflammatory evidence to examine mNGS-clinical discordance (30); and more recent work has applied composite laboratory and bioinformatic filters to reduce contamination and nonspecific mapping (18). Contemporary guidance likewise emphasizes specimen handling, neuroanatomical context, host inflammatory response, and pathogen kinetics (20). Negative controls, background filtering, coverage assessment, and clinical correlation are therefore not novel concepts.

The contribution of the present pathway is to record analytical credibility and clinical attribution as separate judgments for oral-anaerobe signals in low-biomass CSF, building on established control-based and clinical interpretative practices (18, 19, 30). Step 1 documents which control, mapping, and taxonomic evidence is available; Step 2 documents whether host-response, anatomic, source, and temporal findings support or conflict with the signal (**Figure 1**). Discordance prompts reassessment of the particular weak domain and may remain unresolved. The suggested phrases “supports clinical attribution”, “indeterminate attribution”, and “more consistent with background” describe reporting outcomes, not validated diagnostic classes, thresholds, or probabilities. **Figure 2** illustrates contrasting evidence patterns, **Supplementary Table 1** provides intermediate and discordant scenarios with targeted reassessment steps, and **Supplementary Table 2** lists laboratory review questions.

**Figure 1:**
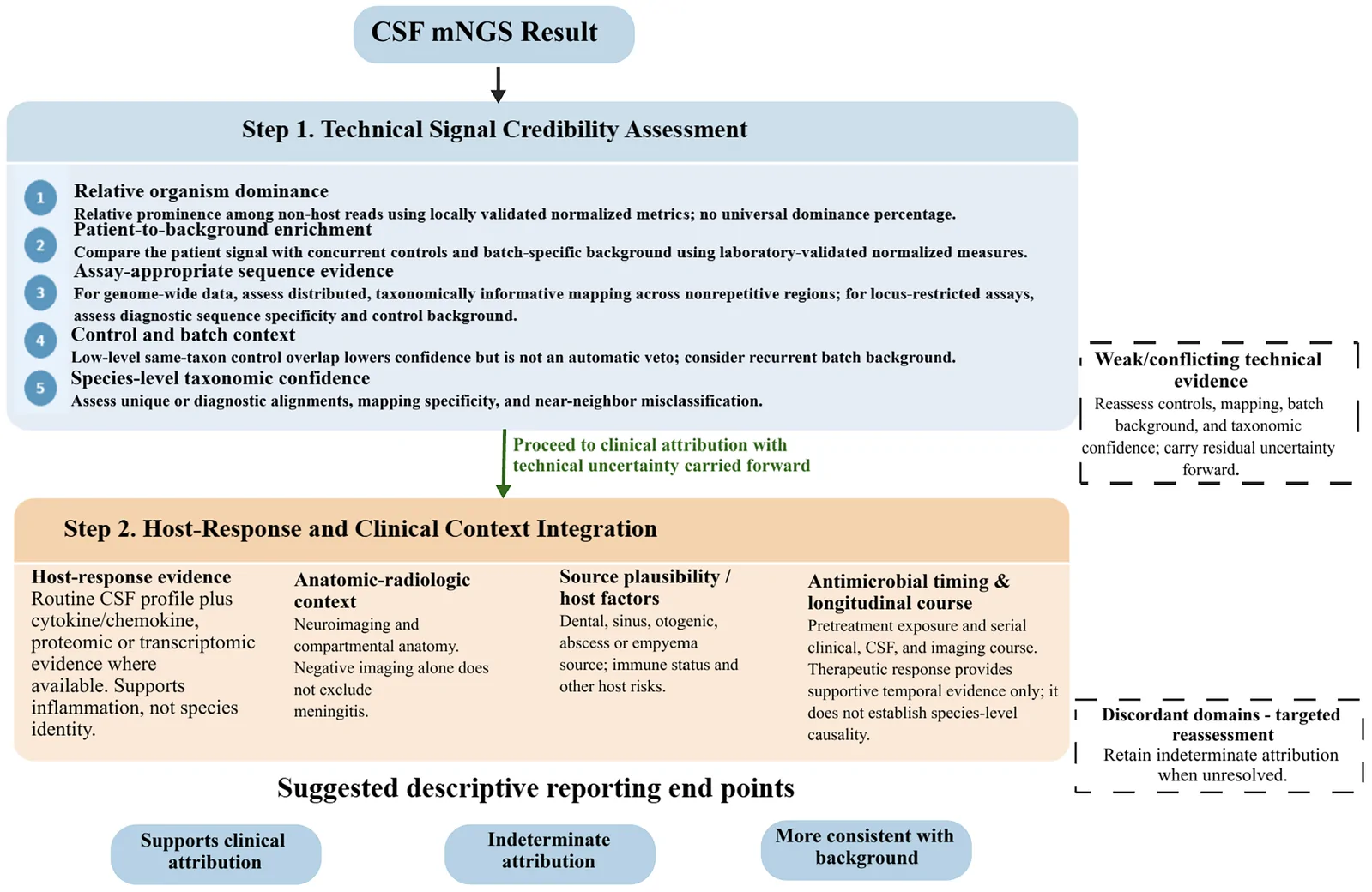
Expert-informed, non-validated pathway for interpreting oral-anaerobe signals detected by CSF mNGS in low-biomass specimens. Step 1 evaluates technical signal credibility through control-relative enrichment, genome-distribution and mapping evidence, batch context, and taxonomic confidence. Step 2 integrates host-response, anatomical, source, antimicrobial-timing, and longitudinal evidence. Discordant findings prompt reassessment of the affected domain; illustrative scenarios, including inflammatory CSF with non-diagnostic initial imaging, are presented in **Supplementary Table 1**. The suggested end points are descriptive reporting phrases, not validated diagnostic classes, numerical thresholds, or estimates of infection probability.

**Figure 2:**
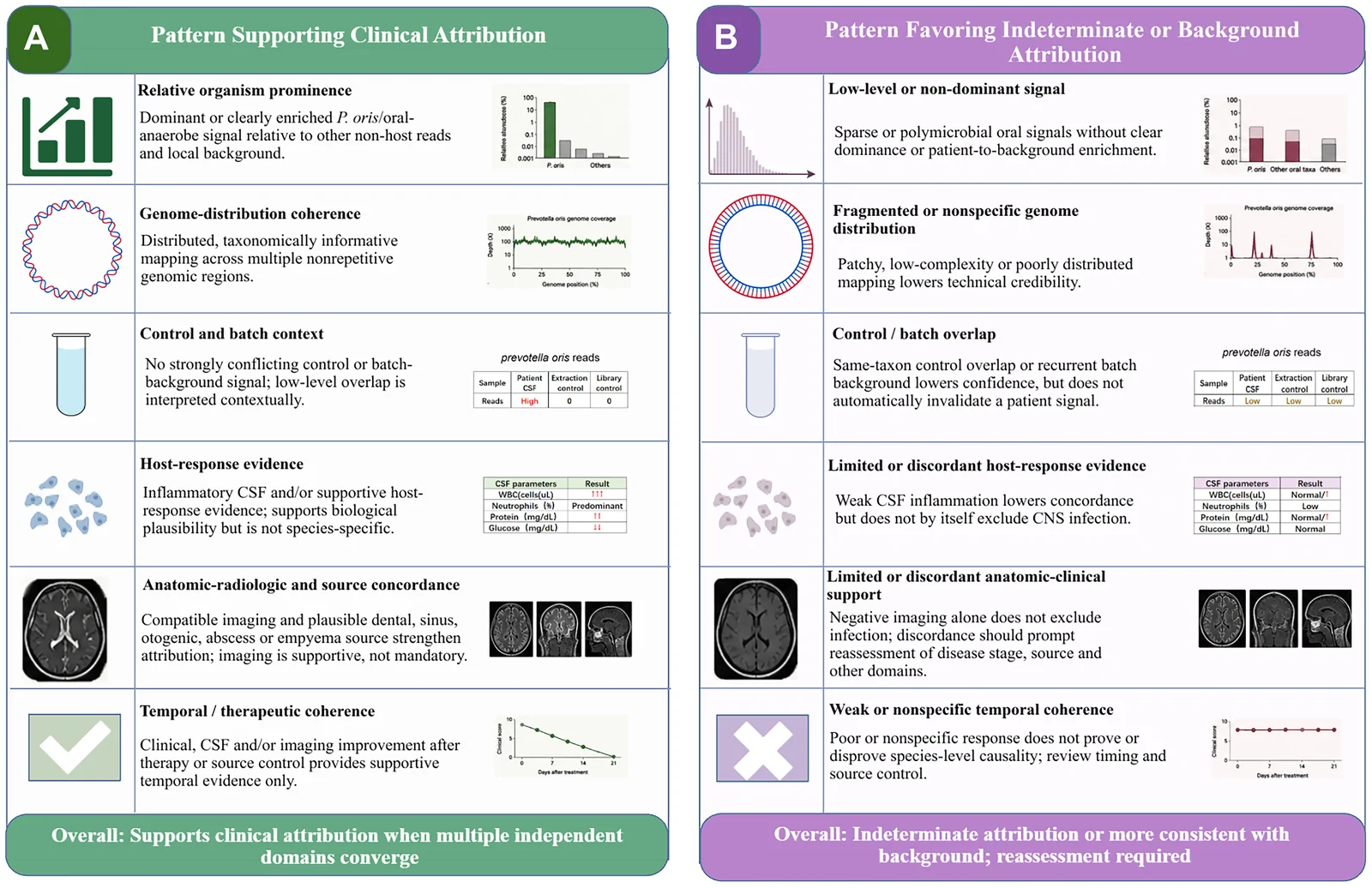
Illustrative evidence patterns for *P. oris* and other oral anaerobes detected in low-biomass CSF. Panel **(A)** depicts a pattern supporting clinical attribution when technically credible microbial evidence converges with independent host-response, anatomic, and source evidence. Panel **(B)** depicts weak technical evidence or discordant clinical domains that require separate reassessment and may result in indeterminate attribution or a finding more consistent with background. A nondiagnostic initial MRI alone does not classify a technically credible signal as background (31). The genome-coverage sketches represent a genome-wide sequencing workflow; locus-restricted assays require sequence evidence appropriate to their design. Therapeutic response is supportive temporal evidence only and does not prove species-level causality. **Supplementary Table 1** provides intermediate and discordant scenarios, including inflammatory CSF with nondiagnostic initial imaging, together with targeted reassessment steps. This expert-informed image is conceptual and non-validated; it provides neither diagnostic thresholds nor estimates of infection probability.

## 5 Clinical management implications

Initial treatment should follow the presenting CNS syndrome and its severity before a CSF species label is accepted as causal. For suspected acute community-acquired bacterial meningitis, WHO recommends intravenous ceftriaxone or cefotaxime, with ampicillin or amoxicillin added for Listeria risk factors and consideration of vancomycin where relevant pneumococcal resistance is prevalent (38). These general meningitis recommendations do not settle management of a concurrent dental or sinus focus, abscess, or empyema; suspected focal suppurative disease requires assessment of anaerobic and polymicrobial coverage and of source control. For community-acquired brain abscess in immunocompetent patients, ESCMID recommends a third-generation cephalosporin plus metronidazole and aspiration or excision whenever feasible; its 6–8-week treatment recommendation is conditional and supported by low-certainty evidence (34). That abscess duration should not be assigned to uncomplicated meningitis on the basis of an mNGS taxon alone.

When mNGS reports *P. oris* or another oral anaerobe, clinicians should pursue culture and susceptibility testing when feasible and avoid automatic narrowing to a single agent. A species-level mNGS call does not provide phenotypic susceptibility data. When a clinically relevant specimen can be obtained, culture and susceptibility testing should inform treatment review; narrowing therapy solely because CSF mNGS reports *P. oris* may also overlook a polymicrobial source or an unresolved species assignment (34, 38). The infected compartment, host factors, polymicrobial possibility, and source control also matter. Dental, sinus, otogenic, abscess, or empyema sources should be actively sought; a systematic review of complicated odontogenic sinusitis found that management commonly required both systemic antimicrobial therapy and intervention directed at the intracranial/orbital complication and the dental or sinus source (39). Therapeutic response can strengthen temporal coherence, but improvement during broad anaerobe-active therapy or after drainage is supportive rather than species-specific proof of causality.

## 6 Discussion, limitations, and future directions

The proposed pathway should be read as an expert-informed organizational model, not as a validated diagnostic algorithm. Its central premise is deliberately modest: technical credibility and clinical attribution are related but distinct questions. That distinction is particularly important for oral anaerobes because biological plausibility can coexist with low-biomass contamination risk. The framework is limited by variation in specimen processing, amplification and enrichment strategies, sequencing workflows, laboratory background profiles, taxonomic databases, and reporting thresholds; these differences also restrict direct comparison of read abundance and sequence distribution across assays (8, 11, 17). Detection of microbial nucleic acid also does not, by itself, establish organism viability (1). The clinical evidence base is also uneven across syndromes and taxa: much of the comparative oral-anaerobe evidence derives from brain-abscess and other intracranial suppurative cohorts (3, 9), whereas *P. oris* CNS evidence remains limited to rare case reports (37), limiting direct extrapolation to isolated meningitis and precluding species-specific diagnostic claims. This narrative search was targeted rather than systematic and may not have captured every eligible publication.

Prospective validation should therefore focus on calibration within specified assay and library designs rather than simply assigning a score: laboratories should assess batch background, normalized abundance, assay-appropriate sequence evidence, sequencing depth, and taxonomic confidence across independent cohorts, while clinical studies should test whether host-response and source/anatomic information improve the reproducibility of attribution. Any future numerical model would require prespecified thresholds and external validation before use for diagnostic classification.

## 7 Conclusions

In culture-negative suspected anaerobic CNS infection, CSF mNGS can add microbiological evidence, but a species-level detection alone cannot establish active infection. *P. oris* illustrates this attribution problem because its oral ecology and reported CNS cases make infection plausible without establishing a distinctive clinical or immune signature. Separating technical signal credibility from clinical attribution may make uncertainty and the need for targeted reassessment clearer in reporting; the pathway remains non-validated and cannot assign an infection probability or replace multidisciplinary clinical judgment.

## References

[1] Su LD Chiu CY Gaston D Hogan CA Miller S Simon DW . Clinical metagenomic next-generation sequencing for diagnosis of central nervous system infections: Advances and challenges. Mol Diagn Ther. (2024) 28:513–23. doi: 10.1007/s40291-024-00727-9 38992308 PMC11660858

[2] Wilson MR Sample HA Zorn KC Arevalo S Yu G Neuhaus J . Clinical metagenomic sequencing for diagnosis of meningitis and encephalitis. N Engl J Med. (2019) 380:2327–40. doi: 10.1056/NEJMoa1803396 31189036 PMC6764751

[3] Wang X Guo X Liu H Wang B Wu J Chen S . Augmented pathogen detection in brain abscess using metagenomic next-generation sequencing: A retrospective cohort study. Microbiol Spectr. (2024) 12:e0032524. doi: 10.1128/spectrum.00325-24 39264158 PMC11448231

[4] Li X Fan M Yue J Xie J Zhang Y Lu X . Metagenomic next-generation sequencing for brain abscess: Improved pathogen detection, targeted antimicrobial therapy, and association with fewer surgical interventions. Infection Drug Resistance. (2026) 19:617362. doi: 10.2147/idr.S617362 42472228 PMC13380248

[5] Salter SJ Cox MJ Turek EM Calus ST Cookson WO Moffatt MF . Reagent and laboratory contamination can critically impact sequence-based microbiome analyses. BMC Biol. (2014) 12:87. doi: 10.1186/s12915-014-0087-z 25387460 PMC4228153

[6] Davis NM Proctor DM Holmes SP Relman DA Callahan BJ . Simple statistical identification and removal of contaminant sequences in marker-gene and metagenomics data. Microbiome. (2018) 6:226. doi: 10.1186/s40168-018-0605-2 30558668 PMC6298009

[7] Karstens L Asquith M Davin S Fair D Gregory WT Wolfe AJ . Controlling for contaminants in low-biomass 16s rrna gene sequencing experiments. mSystems. (2019) 4(4):e00290-19. doi: 10.1128/mSystems.00290-19 31164452 PMC6550369

[8] Olausson J Brunet S Vracar D Tian Y Abrahamsson S Meghadri SH . Optimization of cerebrospinal fluid microbial DNA metagenomic sequencing diagnostics. Sci Rep. (2022) 12:3378. doi: 10.1038/s41598-022-07260-x 35233021 PMC8888594

[9] Al Masalma M Lonjon M Richet H Dufour H Roche PH Drancourt M . Metagenomic analysis of brain abscesses identifies specific bacterial associations. Clin Infect Dis. (2012) 54:202–10. doi: 10.1093/cid/cir797 22144533

[10] Li X Du H Song Z Wang H Long X . Polymicrobial anaerobic meningitis detected by next-generation sequencing: Case report and review of the literature. Front Med. (2022) 9:840910. doi: 10.3389/fmed.2022.840910 35273982 PMC8902384

[11] Simner PJ Miller HB Breitwieser FP Pinilla Monsalve G Pardo CA Salzberg SL . Development and optimization of metagenomic next-generation sequencing methods for cerebrospinal fluid diagnostics. J Clin Microbiol. (2018) 56(9):e00472-18. doi: 10.1128/jcm.00472-18 29976594 PMC6113476

[12] Sung YH Ju YK Lee HJ Park SM Suh JW Kim JY . Clinical performance of real-time nanopore metagenomic sequencing for rapid identification of bacterial pathogens in cerebrospinal fluid: A pilot study. Sci Rep. (2025) 15:3493. doi: 10.1038/s41598-025-87858-z 39875797 PMC11775224

[13] Xie D Liu Y Cong W Qi G Mu C . Pathogen diagnostic value of nanopore sequencing in postoperative central nervous system infections. World Neurosurg. (2025) 199:124090. doi: 10.1016/j.wneu.2025.124090 40379209

[14] Van Dong D Linh LTK Nga NTT Hoan NX Linh NTK Huyen TTT . Evaluating the diagnostic utility of 16s Oxford Nanopore Technology sequencing in patients with central nervous system infections and its usefulness in antimicrobial stewardship. J Infect Dis. (2025) 232:e309–17. doi: 10.1093/infdis/jiaf280 40418729 PMC12349954

[15] Jang Y Kim S Kim N Son H Ha EJ Koh EJ . Nanopore 16s sequencing enhances the detection of bacterial meningitis after neurosurgery. Ann Clin Transl Neurol. (2022) 9:312–25. doi: 10.1002/acn3.51517 35124895 PMC8935320

[16] Hodor P Pope CE Whitlock KB Hoffman LR Limbrick DL McDonald PJ . Molecular characterization of microbiota in cerebrospinal fluid from patients with csf shunt infections using whole genome amplification followed by shotgun sequencing. Front Cell Infect Microbiol. (2021) 11:699506. doi: 10.3389/fcimb.2021.699506 34490140 PMC8417900

[17] Olie SE Staal SL da Cruz Campos AC van de Beek D Brouwer MC . Target enriched metagenomics in cerebrospinal fluid in central nervous system infections. J Infect. (2026) 93:106841. doi: 10.1016/j.jinf.2026.106841 42636981

[18] Cumley N Quick J Brier T Wilkinson S Kent C Hassan-Smith Z . Pathogen detection in central nervous system infections: Moving metagenomic sequencing closer to clinical practice. BMC Infect Dis. (2026) 26(1):1517. doi: 10.1186/s12879-026-13276-9 42277703 PMC13483498

[19] Wilson MR O'Donovan BD Gelfand JM Sample HA Chow FC Betjemann JP . Chronic meningitis investigated via metagenomic next-generation sequencing. JAMA Neurol. (2018) 75:947–55. doi: 10.1001/jamaneurol.2018.0463 29710329 PMC5933460

[20] Waldrop G Reddy SP . Metagenomic next-generation sequencing in central nervous system infections: Clinical strategies, evidence, and best practices. Clin Infect Dis. (2026) 82:S92–9. doi: 10.1093/cid/ciag120 42139092

[21] Waage A Halstensen A Espevik T Brandtzæg P . Compartmentalization of tnf and il-6 in meningitis and septic shock. Mediators Inflammation. (1993) 2:23–5. doi: 10.1155/s096293519300002x 18475498 PMC2365385

[22] Ichiyama T Isumi H Yoshitomi T Nishikawa M Matsubara T Furukawa S . Nf-kappab activation in cerebrospinal fluid cells from patients with meningitis. Neurol Res. (2002) 24:709–12. doi: 10.1179/016164102101200627 12392210

[23] Spanaus KS Nadal D Pfister HW Seebach J Widmer U Frei K . C-X-C and C-C chemokines are expressed in the cerebrospinal fluid in bacterial meningitis and mediate chemotactic activity on peripheral blood-derived polymorphonuclear and mononuclear cells *in vitro*. J Immunol. (1997) 158:1956–64. doi: 10.4049/jimmunol.158.4.1956 9029138

[24] Mook-Kanamori BB Brouwer MC Geldhoff M Ende A van de Beek D . Cerebrospinal fluid complement activation in patients with pneumococcal and meningococcal meningitis. J Infect. (2014) 68:542–7. doi: 10.1016/j.jinf.2013.12.016 24412248

[25] Sharief MK Ciardi M Thompson EJ . Blood-brain barrier damage in patients with bacterial meningitis: Association with tumor necrosis factor-alpha but not interleukin-1 beta. J Infect Dis. (1992) 166:350–8. doi: 10.1093/infdis/166.2.350 1634806

[26] Ye Q Shao WX Shang SQ Shen HQ Chen XJ Tang YM . Clinical value of assessing cytokine levels for the differential diagnosis of bacterial meningitis in a pediatric population. Med (Baltimore). (2016) 95:e3222. doi: 10.1097/md.0000000000003222 27043692 PMC4998553

[27] Bakochi A Mohanty T Pyl PT Gueto-Tettay CA Malmström L Linder A . Cerebrospinal fluid proteome maps detect pathogen-specific host response patterns in meningitis. eLife. (2021) 10:e64159. doi: 10.7554/eLife.64159 33821792 PMC8043743

[28] Ramachandran PS Ramesh A Creswell FV Wapniarski A Narendra R Quinn CM . Integrating central nervous system metagenomics and host response for diagnosis of tuberculosis meningitis and its mimics. Nat Commun. (2022) 13:1675. doi: 10.1038/s41467-022-29353-x 35354815 PMC8967864

[29] Xing Z Jiang H Liu X Chai Q Xin Z Zhu C . Integrating DNA/RNA microbe detection and host response for accurate diagnosis, treatment and prognosis of childhood infectious meningitis and encephalitis. J Transl Med. (2024) 22:583. doi: 10.1186/s12967-024-05370-w 38902725 PMC11191231

[30] Wang J Ye J Yang L Chen X Fang H Liu Z . Inconsistency analysis between metagenomic next-generation sequencing results of cerebrospinal fluid and clinical diagnosis with suspected central nervous system infection. BMC Infect Dis. (2022) 22:764. doi: 10.1186/s12879-022-07729-0 36180859 PMC9523998

[31] Lummel N Koch M Klein M Pfister HW Brückmann H Linn J . Spectrum and prevalence of pathological intracranial magnetic resonance imaging findings in acute bacterial meningitis. Clin Neuroradiol. (2016) 26:159–67. doi: 10.1007/s00062-014-0339-x 25245328

[32] van Soest TM Chekrouni N van Sorge NM Brouwer MC van de Beek D . Bacterial meningitis presenting with a normal cerebrospinal fluid leukocyte count. J Infect. (2022) 84:615–20. doi: 10.1016/j.jinf.2022.02.029 35245581

[33] Shangguan L Xue L Shang J Wang H . The application value of metagenomic next-generation sequencing in community-acquired purulent meningitis after antibiotic intervention. BMC Infect Dis. (2023) 23:683. doi: 10.1186/s12879-023-08672-4 37828452 PMC10571418

[34] Bodilsen J D'Alessandris QG Humphreys H Iro MA Klein M Last K . European Society of Clinical Microbiology and Infectious Diseases guidelines on diagnosis and treatment of brain abscess in children and adults. Clin Microbiol Infect. (2024) 30:66–89. doi: 10.1016/j.cmi.2023.08.016 37648062

[35] Riggio MP Lennon A . Development of a novel pcr assay for detection of Prevotella oris in clinical specimens. FEMS Microbiol Lett. (2007) 276:123–8. doi: 10.1111/j.1574-6968.2007.00926.x 17937671

[36] Zhang WW Ai C Mao CT Liu DK Guo Y . Prevotella oris-caused meningitis and spinal canal infection: A case report. World J Clin cases. (2023) 11:3830–6. doi: 10.12998/wjcc.v11.i16.3830 37383115 PMC10294159

[37] Li C Lv C Yu M Zhang Y Ling L Yue W . A case report of a brain abscess due to Prevotella oris and a review of the literature. BMC Infect Dis. (2023) 23:633. doi: 10.1186/s12879-023-08306-9 37759232 PMC10523687

[38] Who Guidelines Approved by the Guidelines Review Committee . Who Guidelines on Meningitis Diagnosis, Treatment and Care. Geneva: World Health Organization (2025).

[39] Craig JR Cheema AJ Dunn RT Vemuri S Peterson EL . Extrasinus complications from odontogenic sinusitis: A systematic review. Otolaryngol Head Neck Surg. (2022) 166:623–32. doi: 10.1177/01945998211026268 34253072

