# Supplementary material for: Cerebrospinal fluid metagenomic next-generation sequencing for culture-negative anaerobic central nervous system infection involving Prevotella oris: attribution challenges in low-biomass specimens
Source: Front Immunol. 2026 Oct 12;17:1896093. doi: 10.3389/fimmu.2026.1896093 (PMC13696952; doi:10.3389/fimmu.2026.1896093)
Supplement: Supplementary file 1 [file Table1.docx]

**Supplementary Table S1. Illustrative interpretation and targeted reassessment of oral-anaerobe signals detected by CSF mNGS in low-biomass specimens**

| **Illustrative evidence pattern** | **Interpretative implication** | **Targeted reassessment** | **Suggested report wording** |
| --- | --- | --- | --- |
| Technically credible oral-anaerobe signal with inflammatory CSF, compatible imaging, and a plausible source | Convergent independent findings support clinical attribution, without proving species-level causality. | Review assay-appropriate sequence specificity, process-matched controls, alternative pathogens, antimicrobial timing, and culture or lesion sampling where feasible. | “The oral-anaerobe signal supports clinical attribution in the setting of concordant CSF, anatomic, and source evidence; species-level causality remains inferential.” |
| Technically credible oral-anaerobe signal with inflammatory CSF but a nondiagnostic initial MRI | A nondiagnostic MRI alone does not exclude bacterial meningitis. Attribution depends on the remaining technical and clinical evidence. | Recheck the timing and scope of imaging; review controls, taxonomic specificity, competing causes of CSF inflammation, pretreatment, and dental, sinus, or otogenic sources. Consider follow-up imaging if the clinical course continues to suggest focal infection. | “The inflammatory CSF profile and technically credible oral-anaerobe signal support continued assessment of CNS infection despite nondiagnostic initial imaging; attribution remains provisional pending targeted reassessment.” |
| Compatible abscess or empyema imaging with little CSF pleocytosis | Limited lumbar CSF inflammation should prompt assessment of compartment, immune status, and treatment timing. Rare confirmed bacterial meningitis episodes also occur without CSF pleocytosis. Imaging does not establish the species involved. | Review immune status and prior antibiotics; assess the lesion and its source, and obtain lesion material for microbiology when clinically feasible. | “The focal anatomic findings support CNS infection, but the CSF signal alone does not establish that the reported species caused the lesion.” |
| Inflammatory CSF with a weak, nonspecific, or poorly resolved oral-anaerobe signal | Host-response evidence supports inflammation but cannot identify its microbial cause. Species-level attribution remains indeterminate. | Reassess negative controls, batch background, assay-appropriate sequence evidence, near-neighbor classification, and alternative infectious or noninfectious causes. | “CSF inflammation is present, but the available sequence evidence is insufficient to attribute it to the reported oral-anaerobe species.” |
| The same taxon is detected in patient and negative-control libraries | Control overlap lowers confidence but is not an automatic veto. | Compare normalized patient-to-background enrichment, process-matched controls, recurrence across the batch, and sequence evidence appropriate to the assay. | “The patient signal overlaps with control background; clinical attribution remains indeterminate pending control- and sequence-level review.” |
| Multiple oral anaerobes are detected with a plausible head-and-neck source | Polymicrobial infection is possible, but low-biomass contamination and mixed background signals remain alternatives. | Review each taxon separately against controls and sequence evidence; assess source and compartment; seek conventional microbiology from a relevant specimen when feasible. | “The mixed oral-anaerobe signals may be compatible with a polymicrobial source, but their individual clinical significance remains uncertain.” |
| Sparse, non-dominant oral-commensal signals without independent host, anatomic, or source support | The overall pattern may be more consistent with background, particularly when similar signals recur in controls or the batch. | Check normalized abundance, batch recurrence, taxonomic resolution, and whether new clinical evidence changes the interpretation. | “The low-level oral-commensal signals are more consistent with background on the evidence currently available; reassessment is warranted if independent evidence emerges.” |

**Note:** These patterns are expert-informed illustrations, not validated diagnostic categories, a scoring system, or estimates of infection probability. “Supports clinical attribution,” “indeterminate attribution,” and “more consistent with background” are descriptive reporting outcomes. No universal cutoff for read count, relative abundance, patient-to-control enrichment, or genome coverage is implied. Genome-distribution assessment applies only to workflows that generate genome-wide reads; locus-restricted assays require sequence and control checks appropriate to their design. A therapeutic response supplies supportive temporal evidence, not species-specific proof of causality.

**Supplementary Table S2. Operational interpretation principles for assessing the technical credibility of low-biomass CSF mNGS signals.**

| **Technical dimension** | **Suggested operational interpretation** | **Findings that increase credibility** | **Findings that lower credibility** | **Key limitation/evidence** |
| --- | --- | --- | --- | --- |
| **Organism dominance** | Assess relative prominence among non-host microbial reads using a laboratory-validated normalized metric rather than a universal percentage. | The organism remains among the leading microbial signals after normalization. | The organism represents only a minor component among multiple low-level taxa. | Relative metrics are assay dependent; no universal dominance percentage is established. |
| **Enrichment above background** | Compare the patient signal with concurrently processed negative controls and laboratory-specific background using locally validated metrics such as control-derived z scores or normalized patient/control ratios. | Clear patient-to-control or patient-to-background enrichment. | Patient and background signals are similar after normalization. | Published workflows use different validated background models. |
| **Genome-distribution coherence in genome-wide data** | When the assay provides genome-wide reads, examine mapping across distinct, nonrepetitive regions rather than clustering in a few low-complexity or nonspecific loci. | Distributed, taxonomically informative mapping across distinct regions. | Focal or nonspecific mapping with limited sequence diversity. | This criterion cannot be applied unchanged to locus-restricted 16S amplicons; amplification and enrichment effects require workflow-specific interpretation. |
| **Negative-control overlap** | Treat low-level detection of the same taxon in a negative control as a reason to lower confidence, not as an automatic veto. | Patient signal is substantially enriched and independently supported by mapping evidence. | Similar patient/control abundance or recurrent same-batch detection. | Simple removal of every taxon detected in controls can remove genuine low-abundance sequences. |
| **Sequencing-depth normalization** | Prefer normalized metrics for comparisons across libraries with different total sequencing depths. | Enrichment persists after normalization. | Apparent enrichment depends primarily on a larger total read yield. | Raw read counts are sequencing-depth and workflow dependent. |
| **Recurrent contaminants** | Interpret recurrent low-level taxa against laboratory- and batch-specific background profiles. | Patient signal clearly exceeds the recurrent background pattern. | Similar low-level detection repeatedly occurs in blanks or unrelated samples. | Reagent/background composition can vary by kit and batch and becomes more influential at low biomass. |
| **Species-level taxonomic confidence** | Assess whether available unique or diagnostic alignments and complementary classification/mapping checks can distinguish the reported species from near-neighbor taxa. | Multiple informative alignments consistently support the species assignment. | Sparse, repetitive, or nonspecific alignments do not securely resolve species identity. | If the necessary classification information is unavailable, species-level credibility should be considered incompletely assessable rather than assumed. |
| **Assay and library design** | Record the sequencing platform, whether the library is shotgun or target-restricted, and any whole-genome amplification or capture step; apply only metrics validated for that combination. | A patient signal is supported by process-matched controls and sequence evidence appropriate to the assay. | A targeted or amplified signal is interpreted using genome-wide criteria that the assay cannot provide, or by raw-read thresholds transferred from another workflow. | WGA alone may provide little relative pathogen enrichment without host depletion, and target-capture testing may yield unexpected positives requiring interpretation. |

**Note:** These principles are expert-informed operationalizations of published laboratory and bioinformatic evidence and are not validated universal diagnostic thresholds. Laboratories should preferentially apply metrics and cutoffs established for their own validated workflow. Read abundance and sequence-distribution criteria should also be matched to the library design and any amplification or enrichment steps.
